# Plant protein consumption is associated with body mass index among women of reproductive age in Indonesia

**DOI:** 10.3389/fnut.2023.1243635

**Published:** 2023-10-18

**Authors:** Fitra Sistia, Helda Khusun, Judhiastuty Februhartanty

**Affiliations:** ^1^Department of Nutrition, Faculty of Medicine, Universitas Indonesia – Dr. Cipto Mangunkusumo General Hospital, Jakarta, Indonesia; ^2^SEAMEO Regional Centre for Food and Nutrition (RECFON)—Pusat Kajian Gizi Regional Universitas Indonesia, Jakarta, Indonesia; ^3^Faculty of Health Sciences, Universitas Muhammadiyah Prof Dr. HAMKA (UHAMKA), Jakarta, Indonesia

**Keywords:** body mass index, Indonesia, obesity, plant protein, protein consumption, women of reproductive age

## Abstract

**Introduction:**

One of the known determinants of obesity in Southeast Asia countries, including Indonesia, is the nutritional transition, which is indicated by fast changes in food production, dietary habits, and physical activity. With rising incomes, plant protein from grains, tubers, and legumes is gradually being replaced by animal protein from poultry, eggs, dairy, and red meat. This change is identified as a protein transition. Different choices of protein sources in the diet have varying health effects. However, there is limited information on the Asian population on the role of protein consumption on the increasing obesity prevalence. Therefore, this study aimed to investigate the association of protein sources consumption with body mass index (BMI) among women of reproductive age in Indonesia.

**Methods:**

This study used secondary data from the 2018 Indonesia Food Barometer (IFB) conducted using a quantitative cross-sectional survey. A total of 467 Indonesian reproductive-aged women (20–49 years) were included in this study. Dietary intake, including protein consumption, was obtained using 24-h dietary recall. Multiple linear regression was applied to find the association of protein consumption with BMI with a *p*-value <0.05 considered as a significant outcome variable.

**Results:**

The Mean BMI was 25.02 kg/m2, median of animal and plant protein was 28.01 g/day and 25.37 g/day, respectively. Consumption of plant protein was significantly associated with BMI after adjusting for marital status and age (*p*-value = 0.043; R^2^ = 0.080). The quality of plant protein should be considered to prevent obesity problems among women of reproductive age.

## 1. Introduction

The global health challenge recognizes obesity as an increasing health problem among women of reproductive age in low and middle-income countries (LMICs) (1). According to the World Health Organization (WHO) (2) in 2020, about 22% of the world’s adult population (17% of men and 25% of women) were obese. Southeast Asia (SEA) countries exhibit varying prevalence of obesity, ranging from below 10% to nearly 30% (3–5). Indonesia, in particular, experienced a 6.4% increase in obesity between 2013 and 2018 (6, 7), with a higher prevalence among women (29.3%) as compared to men (14.5%). Specifically among women of reproductive age, the prevalence of obesity was 24.7% (6). Being obese is a significant risk factor for non-communicable diseases (8) and it also contributes to adverse maternal and fetal complications (9).

The nutritional transition, characterized by rapid changes in food production, eating habits, and physical activity, is a known predictor of obesity prevalence in SEA countries, including Indonesia (9, 10). As lower and middle-income countries experience rising incomes, there is a shift from plant to animal proteins in their diets, while the overall protein content remains constant (10–12). In contrast, high-income countries are promoting a transition towards plant-based proteins. Thus, protein intake appears to be determined not only by economic factors but also by geography, religion, ethnicity, modernization, and culture (12–14).

Evidence suggests that different types of protein have varying impacts on health. Higher protein consumption, animal-derived proteins, particularly from processed and red meat, has been linked to an increased risk of overweight and obesity (15). In contrast, plant protein has shown positive association with changes in waist circumference and reduced weight (16). Healthy plant-based dietary patterns emphasizing whole grains, fruits/vegetables, nuts/legumes, vegetable oils, and tea/coffee have been associated with lower weight gain, while less-healthy plant items such as fruit juices, potatoes, and refined grains have been consistently linked to increased weight gain (17).

Despite the significance of proteins in dietary guidelines, there is limited research on the association between protein consumption and the rising obesity prevalence in Asian populations. Therefore, this study aims to investigate the association of protein source consumption with body mass index (BMI) among women of reproductive age in Indonesia. By leveraging the 2018 Indonesia Food Barometer (IFB) dataset, this study aims to explore the relationship between protein source consumption and body mass index (BMI) among women of reproductive age in Indonesia.

## 2. Materials and methods

### 2.1. Study design and population

This study utilized available data from the 2018 Indonesia Food Barometer (IFB) study dataset. The IFB employed a quantitative cross-sectional study design and was conducted in six provinces across Indonesia, consist of West Sumatra, Jakarta, West Java, East Java, Bali, and South Sulawesi. Data collection for the IFB took place through in-person interviews conducted between March and July 2018. Subsequently, the present analysis was conducted between November 2022 to April 2023. The present analysis involved 467 women of reproductive age in the IFB dataset, which fulfilled the following criteria, i.e.: women aged 20–49 years and did not have special conditions, such as pregnant and lactating. Women experiencing changes in diet due to illness were excluded in the study. The sample was determined using total sampling, which means that all respondents who meet the inclusion and do not meet the exclusion criteria for this study was included. The amount of sample was already above the required minimum sample size needed, which was calculated to correlate protein consumption and obesity based on the previous study (18).

### 2.2. Socio-demographic characteristics

Socio-demographic variables consist of age, level of education, wealth index, type of residence, marital status, and occupational physical activity. Age was categorized as younger age (20–34 years old) and older age (35–49 years old). Level of education was captured as lower education (primary – lower secondary) and higher education (upper secondary – college). For wealth index, the scores were calculated based on principal component factors analysis with varimax rotation and were split into tertiles, with T1 as the lowest income, T2 is the middle income, and T3 is the highest income. Type of residence was separated into rural and urban area. Marital status was identified into two categories, which were unmarried and married. In addition, occupational physical activity (OPA) was defined as the type of activity that is associated with a job. OPA classification was determined by Steeves et al. (19).

### 2.3. Measurement of body mass index

This study used BMI score as an indicator of obesity. BMI is a straightforward index calculated by dividing an individual’s weight in kilograms by the square of their height in meters (kg/m^2^). This measurement is commonly used to classify overweight and obesity in adults. Data was collected using anthropometric measurement, following a standardized procedure (20). The measurements were taken using a stadiometer to determine the height and a scale to measure weight.

### 2.4. Assessment of dietary intake

Dietary intake data were collected by single 24-h food recall, using multiple sources method (MSM). MSM was used for evaluating nutrient data and calculating usual nutrient intakes from 24-h nutrient data using the cut point method (21). To assess inter-relationship between nutrient intakes of individuals to individual nutritional status, multiple recall should be applied. The MSM enabled to combine 24-h recall data with multiple 24-h recall data from the 20% subjects that was available. Initially, individuals were asked to write down everything they had eaten and drunk the day before the survey, from the time they woke up until they went to bed at night. For each food item, respondents estimated the quantities consumed using a food photograph bool of portion sizes (20). Protein consumption was calculated using a customized version of Nutri-Survey for Windows 2007. The software was manually updated with the latest available version of the Indonesian Food Composition Table from 2017. This table included 1,128 foods, including cooked dishes and menus, categorized into 13 food groups. It provided data on energy, water content (moisture), and 19 different nutrients. For this study, the nutrients of interest were energy (measured in kcal) and protein (measured in grams). For dishes or food not listed in the Indonesian FCTs, the recipe approach was used to calculate the nutrients.

The variable of protein sources consumption in this study consisted of four components, which were total protein intake, animal-based protein consumption, plant-based protein consumption, and the ratio of animal to plant-based protein. Total protein intake is defined as the sum of animal-based and plant-based protein intake based on a single 24-h recall, measured in grams per day (22). Animal-based protein sources consisted of poultry, eggs, milk and dairy products, fish and seafood, red meat, and pork. For plant-based protein, included grains and legumes. Furthermore, the ratio of animal to plant-based protein was calculated as the total gram of animal-based protein divided by the plant-based protein ratio (23). This ratio provides insight into the proportion of protein consumed from animal sources compared to plant sources. Additionally, other dietary intakes, including energy, carbohydrate, and fat, were also assessed using the same 24-h recall method, with measurements reported in grams per day (24). Dietary intake was adjusted by energy using energy residual method. To ensure the quality of the dietary data and minimize potential bias, energy under- and over-reporting was estimated using the predicted total energy expenditure (pTEE) technique (25). This technique applied cut-off to identify cases where participants may have provided inaccurate information about their energy intake. Specifically, individuals whose reported energy intake fell outside the range between 40–160% of the predicted total energy expenditure were flagged for further consideration and analysis.

### 2.5. Statistical analysis

The SPSS version 25 was used for data analysis. Statistically significant differences between groups were analyzed using an independent t-test for two groups and One-way ANOVA for more than two groups. Pearson correlation was conducted to analyze the correlation between protein and other dietary intakes with BMI score. Furthermore, Multiple Linear Regression analysis was applied to the association between protein consumption and other covariate variables with BMI score with a *p*-value <0.05 and 95% CI.

## 3. Results

The distribution of the socio-demographic characteristics and BMI among the subjects is presented in Table 1. The age distribution of the respondents was relatively balanced, with 50.3% categorized as younger age and 49.7% as older age. The majority of the subjects (60%) had attained higher education, resided in urban areas (55.2%), were married (83.3%), and had a low-intermediate level of occupational physical activity (80.9%). The wealth index was divided into tertiles, with 26.1% classified as having a low wealth index, 37.3% a middle wealth index, and 36.6% a high wealth index. The mean of subject’s BMI score was 25.02 kg/m^2^.

**Table 1:** Socio demographic characteristic and BMI score of the subjects.

Variables (*n* = 467)	Mean ± SD	Frequency (*n*)	Percentage (%)
BMI score (kg/m^2^)	25.02 ± 4.58		
Age			
Younger age (20–34 years old)		235	50.3
Older age (35–49 years old)		232	49.7
Level of education			
Lower education		187	40.0
Higher education		280	60.0
Wealth index^a^			
Wealth T1		122	26.1
Wealth T2		174	37.3
Wealth T3		171	36.6
Type of residence			
Rural		209	44.8
Urban		258	55.2
Marital status^b^			
Unmarried		78	16.7
Married		389	83.3
Occupational physical activity^c^			
High		65	13.9
Low-intermediate		402	86.1

a Wealth Index was divided into tertile, with T1 as the lowest income, T2 is the middle income, and T3 is the highest income.

b Marital status was categorized as unmarried (single/divorced) and married (living together or not).

c Occupational physical activities (OPA) defined as the type of activity that is associated with a job. OPA classification was determined by Steeves et al. (19).

Table 2 shows that the subjects had a mean total protein intake of 55.98 g/d. In terms of protein sources, the subjects had a median intake of 28.01 g/day of animal-based protein and 25.37 g/day of plant-based protein. Additionally, the median ratio of animal to plant-based protein consumption was 1.5. The average energy, carbohydrate, and fat intakes were 1584.76 kcal/day, 205.10 g/day, and 59.70 g/day, respectively.

**Table 2:** Protein and other dietary intake of the subjects.

Variables	Mean ± SD	Median (Min – Max)	E (%)
Total protein intake (g/d)	55.98 ± 10.75		14.9
Animal-based protein consumption (g/d)		28.01 (0.00–103.56)	7.76
Plant-based protein consumption (g/d)		25.37 (5.80–70.27)	6.59
Ratio of animal-based to plant-based protein		1.50 (0.00–7.43)	
Energy intake (kcal)	1584.76 ± 371.97		
Carbohydrate intake (g/d)	205.10 ± 34.69		54.44
Fat intake (g/d)	59.70 ± 12.21		35.76

The association between protein consumption and BMI among women of reproductive age, after adjusting for covariate variables, is presented in Table 3. The analysis was conducted separately for total protein intake, animal-based and plant-based protein consumption, and the ratio of animal to plant-based protein ratio, to understand each association with BMI score. The initial covariate-adjusted in the analysis were carbohydrate intake, fat intake, age, level of education, marital status, and occupation physical activity (Supplementary Tables S1, S2). However, further analysis found that age and marital status significantly confounded the association between protein consumption and BMI.

**Table 3:** Association of protein consumption with BMI among women reproductive age in Indonesia.

Variables	BMI scores			
	*β*	SE	95% CI	*p*-value^*^
**Model 1** ^a^				
**Constant**	10.146	7.736	−5.057 – 25.349	0.190
Age	1.364	0.429	0.521–2.207	**0.002**
Marital Status	1.850	0.151	0.603–3.099	**0.004**
Total protein intake (g/d)	0.043	0.028	−0.011 – 0.098	0.108
**Model 2** ^b^				
**Constant**	16.960	6.351	4.479–29.441	0.008
Age	1.349	0.429	0.507–2.192	**0.002**
Marital Status	1.832	0.635	0.584–3.080	**0.004**
Plant-based protein consumption (g/d)	0.052	0.026	0.002–0.102	**0.043**
Animal-based protein consumption (g/d)	0.009	0.018	−0.027 – 0.045	0.620
**Model 3** ^c^				
**Constant**	22.830	5.265	12.483–33.177	<0.001
Age	1.364	0.429	0.522–2.207	**0.002**
Marital Status	1.830	0.636	0.581–3.079	**0.004**
Ratio animal to plant-based protein	−0.335	0.208	−0.745 – 0.074	0.108

a Total protein intake and BMI score adjusted with sociodemographic characteristic and other dietary intake.

b Animal protein, plan protein, and BMI score adjusted with sociodemographic characteristic and other dietary intake.

c Ratio of animal to plant-based protein and BMI score adjusted with sociodemographic characteristic and other dietary intake.

*Multiple linear regression with enter method (sig. *p*-value < 0.05), printed bold.

The study found that total protein intake did not show a significant association with BMI score, after adjusting for confounding variables. However, there was a tendency indicating a positive correlation, suggesting that higher protein intake results in a higher BMI score. Similarly, the association between animal-based protein consumption with BMI was not significant, indicating no link between animal protein and BMI in this study. In contrast, the plant-based protein was found to be significantly associated with BMI score, suggesting higher plant protein consumption is linked with higher BMI. In terms of animal-to-plant-based ratio, there was no significant association with BMI score among women of reproductive age after adjusting with the covariate variables.

All multivariate analyses in Table 3 show that BMI score was associated with age and marital status among women of reproductive age. Older age and married women were shown to be associated with higher BMI. All models explained only 8% of the variation of BMI among women of reproductive age, indicating that there were more factors other than those used in the study to explain BMI variation among women of reproductive age.

## 4. Discussion

The study population represented women of reproductive age in Indonesia, which characteristics were almost similar to the Indonesian population according to the 2017 Indonesia Demographic and Health Survey, 42% were categorized as younger age, 72% were married, 52% resided in the urban area, more than half women reproductive age had upper middle wealth index. However, a different characteristic was shown in the level of education, only 42% women of reproductive attend higher education (26). Prior study had shown that women have higher risk of obesity compared to man (27, 28). Obesity among women of reproductive age is rising despite maternal undernutrition still being prevalent, especially in LMICs. Furthermore, the consequences of obesity among obese women not only lead to several adverse maternal but also fetal complications during pregnancy, delivery, and postpartum (9).

The subjects’ mean total protein intake was 55.98 g/d, which was consistent with earlier study (13, 29), but slightly below the recommended protein daily values for Indonesian women of 60 g/d (30). The average energy intake of the subjects was 1584.76 kcal/day, consistent with previous research, which found that women of reproductive age consume approximately 1,500 kcal per day (22, 31). Dietary intake of this study was adjusted by energy using energy residual method. Energy adjustment should be applied to separate the effect of energy intake from specific nutrient in relation with the diseases or nutritional status (32).

The average eating pattern in Indonesia is characterized by more dependence on a single staple. Non-starchy foods accounted for 30% of total dietary energy intake, substantially below the global average of 50% (33). However, the findings of this study have shown a slightly higher median intake of animal compared to plant protein with the ratio of animal to plant protein is 1.5. It indicated a shift in protein consumption among Indonesian. In 2014, the average consumption of protein was 53.81 grams, which rose by 8.17 grams in 2020 become 61.98 grams (34). In terms of the sources of protein, it also reported that there was an overall increase in fish and seafood, meat, and egg consumption. Contrary, there was a reduction in cereal consumption (34). This finding was supported by a longitudinal analysis of food expenditures, which found lower expenditure shares for staple foods and higher expenditure shares for meat, eggs, and milk products (35). Majority of animal sources come from poultry as well as fish and seafood. Similar to previous reports, Indonesia has one of the highest fish consumption rates in the world, as well as a diversity of high-protein soy products such as *tahu* (tofu) and tempeh. Meat and dairy consumption are modest by global standards but vary by cultural group and are increasing as income rise (36). Grains were most consumed food sources among plant-based protein compared to legumes. The prior study also shows a similar result on protein food group consumption (13).

This study found that total protein intake and animal-to-plant protein sources ratio had no association with BMI. Previous studies showed inconsistency in the associations between protein intake and obesity indices. Similar to the current result, some studies showed no associations between protein intake and obesity (16, 37). However, other research found association of protein with obesity (23, 38–40). Other studies even showed that higher-protein diets (1.0–1.5 g/kg body weight) were associated with lower BMI and waist circumference. The potential health effects of higher-protein diets appear to be more pronounced in overweight individuals than in normal-weight and obese individuals (39). The result explained that protein intake above the acceptable macronutrient distribution range can increase risk factors for high WC among women of childbearing age (38). Different findings of protein and obesity could be also due to the different proportions of animal protein and plant protein consumption (23). It has been suggested that a certain amount of protein is required for maintaining normal body weight and waist circumference, hence up to this threshold, only the quantity of proteins is beneficial.

This study further found no significant correlation between animal-based protein intake and BMI score among women of reproductive age in Indonesia. Similar to total protein intake, there are inconsistent results regarding animal protein intake and obesity. Some studies showed no association (16), some reported found a positive associations (41), and some showed a negative association between animal protein consumption with obesity (42, 43). Nonetheless, the rise in animal-source food products has both positive and adverse health effects. On the one hand, a few extra grams of animal-sourced foods can significantly improve the nutrition problems, such as undernutrition and micronutrient deficiency in developing countries. On the other hand, excessive consumption of animal-sourced foods (>18% of total energy) is associated with high saturated fat intake and higher mortality (24). Animal proteins are often accompanied by some quantities of saturated fats and cholesterol which might have deleterious effects on health (44).

In contrast to the result on animal protein, the study showed that plant-based protein consumption was significantly correlated with higher BMI among women of reproductive age in Indonesia after being adjusted by other covariate variables. In contrast, most of the previous studies consistently showed an inverse association between plant protein and obesity. Every 1-gram increase in plant protein was related to a 0.046 kg decrease in fat mass. Overall, a 19.2 g increase in plant protein in the vegan group was related to a 0.88 kg reduction in fat mass. The decrease in fat mass was also linked to a higher diet of plant protein, such as vegetables, grains, legumes, and fruits, as well as a lower diet of animal protein (18). Differences in weight gain distinguish for certain foods and beverages might be attributed to varied portion sizes, eating behaviors, satiety effects, or displacement of other foods. Consumption of various plant-based diet indices was linked to diverse degrees of weight gain (45).

The positive association between plant protein consumption and obesity in this study may be related to the quality of food sources of protein. In Indonesia, the main contributor of plant protein was grain (13, 34, 36). Previous research found that a 1-SD rise in an unhealthy form of a plant-based diet index (emphasizing refined grains, potato/fries, sweets, and sweetened drinks/juices) was linked to a 0.36 kg greater weight gain (45). Less-healthy plant foods, such as fruit juices, potatoes, and refined grains, have been consistently linked to an increased risk of weight gain in the unhealthful pro-vegetarian diet pattern (17).

Moreover, different protein sources also vary in amino acids in their ability to either prevent or induce obesity (46). Some research has reported that amino acids, including branched-chain amino acid (BCAA), tryptophan, and glutamate, can promote obesity (47–49). Amino acids such as glutamate are well known can increase obesity risk (47, 48). The positive correlation of glutamate with the obesity-related indices may be related to the regulation of food intake. Glutamate is abundant in wheat protein. Excess wheat consumption may have resulted in an increase in glutamate levels as well as an increase in BMI, visceral fat, and subcutaneous fat area in certain participants (47). Obesogenic amino acid tryptophan is also found in some plant sources, such as dried fruits, nuts, and oats. Furthermore, gut bacteria modulate the effect of plant protein on BMI by promoting appetite suppression. Gut bacteria metabolize choline and L-carnitine to create trimethylamine (TMA), which is then oxidized to produce trimethylamine-N-oxide (TMAO). TMAO has been linked to the development and risk of atherosclerosis (50). Plant-based foods such as avocado and legumes contain trace levels of L-carnitine. On the other hand, while red meat and eggs are high in choline, plant-based meals, including soybeans, potatoes, and most legumes, are also high in choline.

Protein is an essential component in the dietary guideline. Besides the quantity, the quality of protein sources should be considered. Even though some research shows a protective association between plant protein with BMI score, it is also possible that not all plant-based dietary patterns have beneficial effects on body weight. This finding provides specific information regarding the association between the consumption of different protein sources (i.e., plant vs. animal sources) with nutritional status among women of reproductive age, as similar studies are lacking in Indonesian settings. Understanding different outcomes on the nutritional status resulting from different protein sources is beneficial to design specific dietary recommendations. The present study did not separate the sources of protein based on the food groups, e.g., whole grains vs. refined grains, and processed vs. unprocessed meat as one of the limitations. Besides, information on the food preparation and cooking method is lacking in this study. Moreover, the clinical limitations of BMI should be considered. BMI is a surrogate measure of body fatness because it is a measure of excess weight rather than excess body fat. Further research on protein sources consumption with other nutritional status measures may be considered and focus on specific protein food groups to understand more the contribution of the food groups to the nutritional status.

## 5. Conclusion

In conclusion, high consumption of plant-based protein is associated with higher BMI scores. For future recommendations, the quality of plant protein should be considered besides the amount. Women of reproductive age should be encouraged to increase their intake of healthy plant foods while reducing their intake of less-healthy plant food (such as refined grains, potato/fries, sweets, and sweetened drinks/juices) for improved health outcomes. Furthermore, the government needs to improve health promotion and education activity regarding dietary intake, food choices, and obesity.

## Data availability statement

The data used for this study was obtained from the 2018 Indonesia Food Barometer Study from SEAMEO RECFON. Data is available for public use by contacting the data owner. Requests to access these datasets should be directed to research@seameo-recfon.org.

## Ethics statement

The study was approved by the Ethics Committee of the Faculty of Medicine, Universitas Indonesia – Cipto Mangunkusumo Hospital with a letter numbered KET-162/UN2.F1/ETIK/PPM.00.02/2023 for secondary data analysis in which written informed consent for participation was not required. However, written informed consent was obtained from all participants in the primary data collected through the 2018 Indonesian Food Barometer (Ethical Clearance Number: 927/UN2.F1/ETIK/2017).

## Author contributions

FS, HK, and JF conceived and designed the study. HK and JF involved in the primary data collection of IFB 2018, which was used for this study. FS analyzed the data. FS, HK, and JF interpreted the data. FS drafted the manuscript. All authors contributed to the article and approved the submitted version.
