# Supplementary material for: Plant protein consumption is associated with body mass index among women of reproductive age in Indonesia
Source: Front Nutr. 2023 Oct 18;10:1243635. doi: 10.3389/fnut.2023.1243635 (PMC10622764; doi:10.3389/fnut.2023.1243635)
Supplement: Supplementary file 1 [file Table_1.docx]

**Supplementary Materials**

**Supplementary Table 1. Association between Socio Demographic Characteristic and BMI Score among Women of Reproductive Age in Indonesia**

| **Variables** | **BMI Scores** | | | | |  |
| --- | --- | --- | --- | --- | --- | --- |
|  | **n** | **Mean ± SD** | **95% CI** | **p-value** | **p-value between group** | |
| **Age**  Younger age  Older age | 235  232 | 24.10 ± 4.74  25.95 **±** 4.23 | (-2.66 – -1.02) | <0.001^a^ | |  |
| **Level of Education**  Lower education  Higher education | 187  280 | 25.48 **±** 4.59  24.72 **±** 4.56 | (-0.08 – 1.61) | 0.077^a^ | |  |
| **Wealth Index**  Wealth T1  Wealth T2  Wealth T3 | 122  174  171 | 24.76 ± 4.16  24.95 ± 4.63  25.28 ± 4.82 |  | 0.615^b^ | | 1.000  1.000 |
| **Type of residence**  Rural  Urban | 209  258 | 24.89 ± 4.61  25.13 ± 4.56 | (-1.08 – 0.59) | 0.574^a^ | |  |
| **Marital Status**  Unmarried  Married | 78  389 | 22.81 ± 4.85  25.47 ± 4.40 | (-3.75 – -1.57) | <0.001^a^ | |  |
| **Occupational physical activity**  High  Low-Intermediate | 65  402 | 23.17 ± 4.15  25.32 ± 4.58 | (-3.35 – -0.97) | <0.001^a^ | |  |

^a^Independent T-test;(sig. p-value<0.05)

^b^One-way ANOVA;(sig. p-value<0.05)

**Supplementary Table 2. Association between Protein Sources Consumption and Other Dietary Intake with BMI Score among Women of Reproductive Age**

| **Variables** | **BMI Scores** | | | |
| --- | --- | --- | --- | --- |
|  | **r** | **p-value^1^** | **r** | **p-value^2^** |
| Total protein intake (g/d) | 0.024 | 0.608^a^ | 0.023 | 0.625 |
| Animal-based protein consumption (g/d) | -0.077 | 0.095^a^ | -0.079 | 0.088 |
| Plant-based protein consumption (g/d) | 0.124 | **0.007^a^** | 0.117 | **0.012** |
| Ratio of animal-based to plant-based protein | -0.120 | **0.010^a^** | -0.121 | **0.009** |
| Energy intake (kcal) | -0.058 | 0.213^a^ |  |  |
| Carbohydrate intake (g/d) | 0.061 | 0.186^a^ | 0.076 | 0.101 |
| Fat intake (g/d) | -0.066 | 0.157^a^ | -0.073 | 0.113 |

^1^Adjusted model with energy residual method

^2^Partial correlation adjusted by energy

^a^Spearman Correlation;(sig. p-value<0.05, printed bold)
